# Interaction behaviors and structural characteristics of zein/NaTC nanoparticles

**DOI:** 10.1039/c9ra00005d

**Published:** 2019-02-15

**Authors:** Xiaoyong Wang, Min Fan

**Affiliations:** School of Chemistry & Molecular Engineering, East China University of Science and Technology Shanghai 200237 China xiaoyong@ecust.edu.cn +86-21-64252012

## Abstract

Bile salts are biosurfactants distributed in the human gastrointestinal tract, which can significantly influence the structure and functions of orally administrated components. This work has studied the interaction and conformation changes of zein with sodium taurocholate (NaTC) in the formation of zein/NaTC nanoparticles. When the NaTC concentration (*C*_NaTC_) increases from 0 to 0.24 g L^−1^, the particle size of zein/NaTC nanoparticles decreases from 97 to 76 nm, but markedly increases from 76 to 137 nm as *C*_NaTC_ increases from 0.24 to 0.4 g L^−1^. At *C*_NaTC_ = 0–0.24 g L^−1^, the sharply decreased zeta potential of zein/NaTC nanoparticles suggests that NaTC monomers electrostatically bind with zein molecules to form zein/NaTC complexes, which have high steric repulsion and thus aggregate into smaller zein/NaTC nanoparticles. Nevertheless, at *C*_NaTC_ = 0.24–0.4 g L^−1^, the less changed zeta potential of zein/NaTC nanoparticles together with the surface tension result suggests that NaTC dimers formed on zein polypeptide chains due to the hydrophobic interaction cause zein/NaTC complexes to undergo more aggregation into larger zein/NaTC nanoparticles. Compared to little changes in the secondary and tertiary structures of zein molecules at *C*_NaTC_ = 0–0.24 g L^−1^, the absorption, fluorescence, and circular dichroism measurements disclose that the addition of NaTC above 0.24 g L^−1^ can greatly unfold the compact structure of zein molecules with decreased α-helix content.

## Abstract 2

Bile salts are biosurfactants distributed in the human gastrointestinal tract, which can significantly influence the structure and functions of orally administrated components.

## Graphical abstract

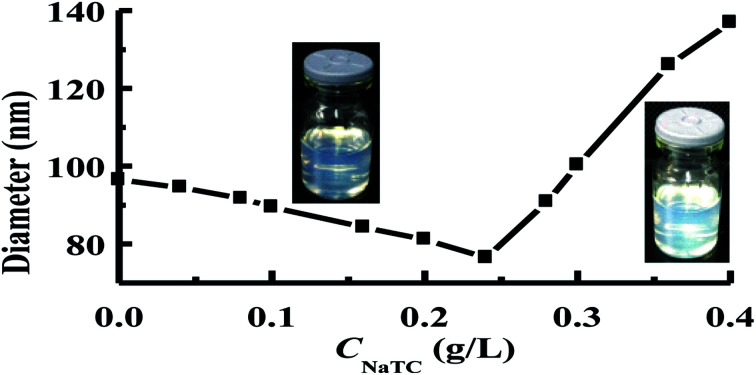

## Introduction

The accurate description of the interaction between proteins and surfactants has important value for diverse phenomena from industrial processes to biological systems.^1^ The interaction of ionic surfactants with proteins may be started by the electrostatic force between the charged headgroups of surfactants and the oppositely charged amino acid residues of proteins, which can be reinforced by the hydrophobic interaction between the alkyl chain of surfactants and the hydrophobic groups of proteins.^2–4^ The subsequently formed protein/surfactant complexes and protein/surfactant nanoparticles can cause conformational changes of proteins, which is significantly dependent on the surfactant structures and features of proteins.

Zein is a plant protein isolated from maize kernels that has a variety of unique characteristics.^5,6^ Amino acid composition analysis shows that zein has a large proportion (>50%) of nonpolar amino acids (leucine, proline, alanine, and phenylalanine), which makes zein insoluble in water but soluble in 60–90% ethanol solution. Owing to the highly inherent hydrophobicity, zein can be easily constructed into zein nanoparticles, which have been used to encapsulate, protect, deliver drug and bioactive molecules.^7^ However, the extensive aggregation of zein may be occurred at some environmental conditions such as pH close to the isoelectric point. Patel *et al.*^8^ and McClements *et al.*^9^ found that the addition of surfactants as the stabilizer can effectively inhibit zein aggregation for the control of particle size of zein nanoparticles. They thought that the stability effect of surfactants on zein nanoparticles is a result of reduced surface hydrophobicity, increased electrostatic and steric repulsions. Recently, we have investigated zein/Tween-20 nanoparticles using the fluorescence and circular dichroism measurements, and demonstrated that the secondary and tertiary structures of zein molecules can experience significant changes during the formation of zein/Tween-20 nanoparticles.^10^

The fates of protein nanoparticles and encapsulated bioactive molecules are possibly challenged by bile salts in human gastrointestinal tract. Bile salts are a group of biosurfactants produced in the liver and play an important role in the digestion and absorption of cholesterol and lipids in the small intestine.^11^ Remarkably different from general aliphatic surfactants with a linear hydrocarbon chain and a hydrophilic headgroup, bile salts have a rigid steroid backbone with methyl groups on the convex hydrophobic surface and hydroxyl groups on the concave hydrophilic surface.^12,13^ The study from Cremers *et al.* revealed that bile salts are effective protein-unfolding reagents, which can increase the aggregation sensitivity of numerous soluble proteins both *in vitro* and *in vivo*.^14^ For example, they found that the addition of bile salts can cause widespread unfolding and aggregation of cytosolic proteins in bacteria. In the study on the solubilization of membrane lipids called the membrane damaging effect,^15^ Begley *et al.* observed that the detergent action of bile salts may alter the conformation of membrane proteins resulting in their misfolding or denaturation.^16^ However, so far, there is no report regarding the details of the interaction behaviors of bile salts with zein molecules and zein nanoparticles.

The present work is aimed to investigate the interaction and conformational behaviors of zein with sodium taurocholate (NaTC) in the formation of zein/NaTC nanoparticles. NaTC is the most abundant bile salt in the human gallbladder, which has a rigid steroid backbone with three methyl groups on the hydrophobic surface, three hydroxyl groups on the hydrophilic surface, and one negatively charged sulfonate group. At a fixed zein concentration of 0.5 g L^−1^, zein/NaTC nanoparticles were prepared by the antisolvent precipitation method upon changing NaTC concentrations at pH 4. Scanning electron microscopy was used to study the morphology of zein/NaTC nanoparticles. The size and charge of zein/NaTC nanoparticles were characterized by dynamic light scattering and zeta potential measurements, respectively. Surface tension method was used to monitor the surface activity of zein/NaTC nanoparticles at different NaTC concentrations. Following the measurements of UV-Vis absorption, fluorescence, and circular dichroism, the effects of added NaTC on the secondary and tertiary structures of zein molecules in zein/NaTC nanoparticles have been further investigated.

## Experimental section

### Materials

Zein (protein content ≥ 97%) and sodium taurocholate (NaTC, purity ≥ 97%) were purchased from Sigma-Aldrich Chemical Company. Absolute ethanol (purity ≥ 99.7%) was obtained from Shanghai Titan Scientific Company. All other chemical reagents used were of analytical grade, and water was double distilled.

### Preparation of zein/NaTC nanoparticles

Zein/NaTC nanoparticles were prepared base on the antisolvent precipitation method.^10^ Briefly, zein powder was added into 80% ethanol/water solution and stirred for 30 min to ensure dissolution. A syringe was used to inject zein solution into pH 4 water containing different NaTC concentrations. The resulting mixed dispersion was continually stirred for another 30 min, and then ethanol was evaporated using a rotary evaporator. To compensate for the loss of ethanol, the same volume of water (pH 4) was added to get the solution of zein/NaTC nanoparticles. The amount of zein was kept at 0.5 g L^−1^ in the final samples, while the concentration of NaTC is varied from 0 to 0.6 g L^−1^.

### Turbidity measurement

Turbidity measurement was carried out with a Shimadzu UV-1800 spectrometer at 25 °C. The turbidity of solution of zein/NaTC nanoparticles was monitored by UV absorbance at 500 nm. A cuvette with 1 cm path length was used. All experiments were performed in triplicate.

### Scanning electron microscopy (SEM)

The microtopography of zein/NaTC nanoparticles was characterized by scanning electron microscopy (SU1510, Hitachi, Japan). Before scanning, the samples were sprayed with a gold layer for 5 min in vacuum and placed in the electron microscope with an acceleration voltage of 5.0 kV to capture images.

### Particle size and zeta potential measurements

The particle size and zeta potential of zein/NaTC nanoparticles were determined using a Malvern Zetasizer Nano ZS (Malvern Instruments, London, England). While the particle size was controlled *in situ* by dynamic light scattering, the zeta potential was determined based on the Smoluchowski relation of the ionic mobility with the surface charge. The samples were diluted with pH 4 water before the measurement to avoid the multiple scattering effect. All measurements were carried out at 25 °C.

### Surface tension measurement

The surface tensions of zein/NaTC nanoparticles were measured by a Dataphysics model DCAT11 tensiometer (Sartorius, Goettingen, Germany) using the Wilhelmy plate method at 25 °C. Before each measurement, the plate was firstly rinsed with double distilled water and then burned to red. For comparability of equilibrium surface tension, the measurements were stopped when the standard deviation of the surface tension was less than 0.03 mN m^−1^.

### UV-Vis absorption measurement

The absorption measurement was made with a Shimadzu UV-1800 spectrophotometer at 25 °C. The absorption spectra of zein molecules in zein/NaTC nanoparticles were taken in the wavelength region of 250–400 nm.

### Steady-state fluorescence measurement

Steady-state fluorescence measurement for zein/NaTC nanoparticles was performed on a Shimadzu RF-5301 spectrofluorophotometer at 25 °C. The intrinsic fluorescence of zein molecules in zein/NaTC nanoparticles was recorded from 285 to 400 nm with an excitation wavelength of 278 nm. The slit widths of excitation and emission were set at 3 and 5 nm, respectively.

### Circular dichroism measurement

Circular dichroism (CD) spectra of zein molecules in zein/NaTC nanoparticles were measured on a Chirascan automatic recording spectrophotometer at 25 °C. The CD spectra in a range of 195–250 nm were recorded with a quartz cell of 1 mm path length at a scan speed of 120 nm min^−1^. The results were expressed as ellipticity in millidegrees (mdeg). The α-helix content of zein molecules was analyzed using CDNN, a deconvolution program for protein secondary structure analysis from CD data (Chirascan).

### Statistical analysis

Data are presented as mean and standard deviations. For all measurements, a minimum of three replicates was taken for data analysis.

## Results and discussion

### Formation of zein/NaTC nanoparticles

**Fig. 1:**
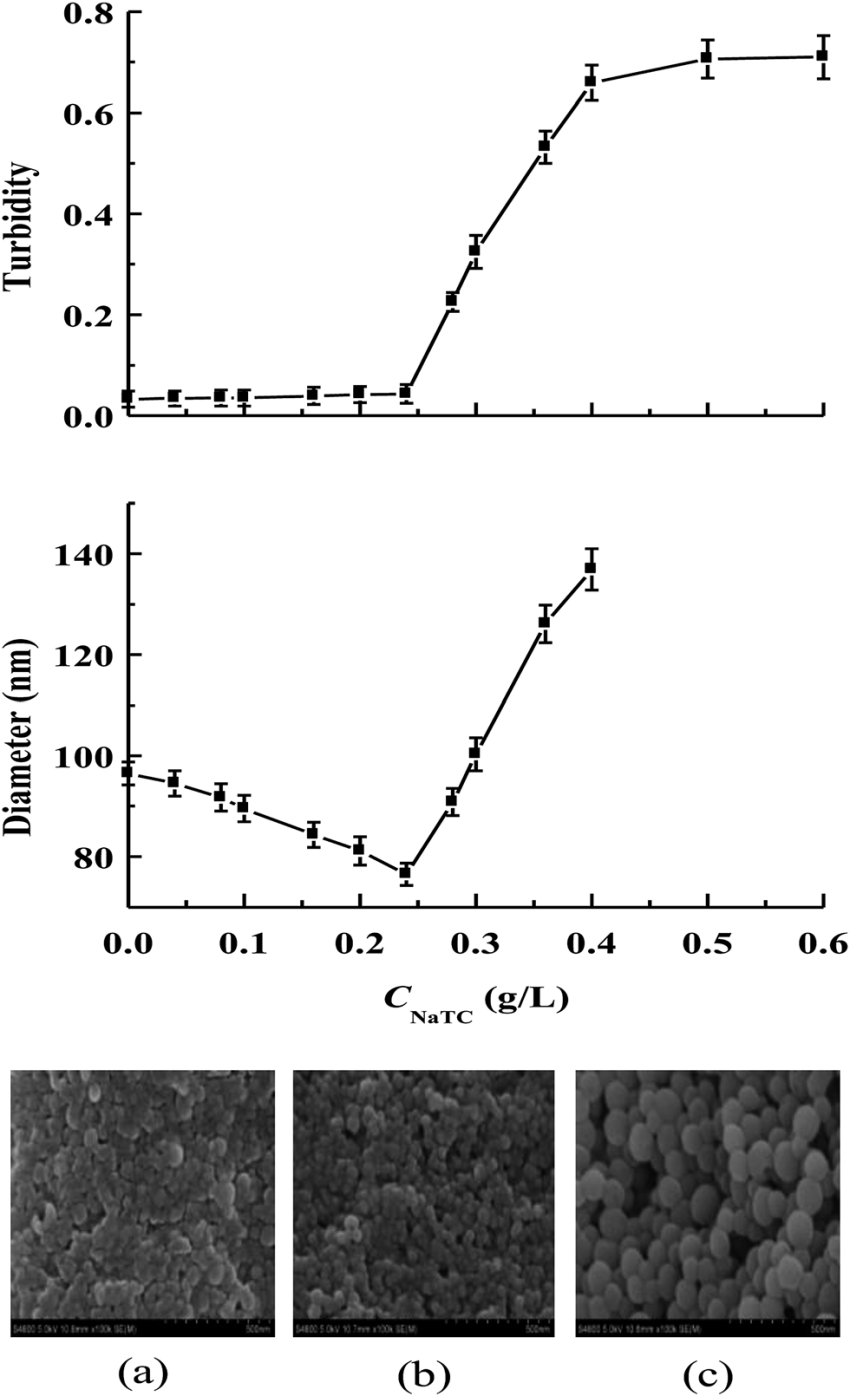
Turbidity and particle size of zein/NaTC nanoparticles as a function of NaTC concentration. The turbidity is reported as the absorbance of the sample of zein/NaTC nanoparticles at 500 nm. Attached SEM pictures of representative zein/NaTC nanoparticles: (a) 0 g L^−1^ NaTC; (b) 0.16 g L^−1^ NaTC; (c) 0.36 g L^−1^ NaTC.

Zein is insoluble in pure water but can be dissolved in the mixture of ethanol and water at an ethanol concentration of 60–90%.^5^ The antisolvent precipitation method has been used to prepare zein/NaTC nanoparticles at pH 4, by injecting zein dissolved in 80% ethanol/water mixed solution into the water phase containing different amounts of NaTC. Fig. 1 gives the turbidity and particle size of zein/NaTC nanoparticles as a function of the concentration of NaTC (*C*_NaTC_), together with SEM images of representative zein/NaTC nanoparticles. Without the addition of NaTC, the sample of plain zein nanoparticles is a relatively transparent solution with the turbidity close to zero, and the particle size of plain zein nanoparticles is 97 nm. Using small-angle X-ray scattering, Matsushima *et al.* observed that zein molecules exist as unique asymmetric particles of 13 nm in length with an axial ratio of 6 : 1 in ethanol solution.^17^ They proposed that zein molecules have an elongated prism-like shape with hydrophobic sides, while the top and bottom containing hydrophilic amino acid residues. After dispersed in the water phase, the strong hydrophobic attraction between the exposed nonpolar groups on the hydrophobic sides of zein molecules is the dominant force for the aggregation into plain zein nanoparticles.^18^ On the other hand, zein molecules carry positive charges totally, due to the protonation of amino groups at pH 4 which is lower than the isoelectric point (pH 6.2) of zein.^5^ Therefore, the positively charged groups together with the hydrophilic amino acid residues of zein molecules may inhibit the extensive aggregation and subsequently contribute to the stability of plain zein nanoparticles.

When *C*_NaTC_ increases from 0 to 0.24 g L^−1^, the particle size of zein/NaTC nanoparticles can be seen to gradually decrease from 97 to 76 nm. SEM pictures also give the evidence for better dispersion of zein/NaTC nanoparticles at these NaTC concentrations than plain zein nanoparticles. Surfactants, such as Tween and sodium dodecyl sulfate (SDS), were reported to have the ability to reduce the particle size of zein nanoparticles,^9,19^ which is ascribed to the decreased hydrophobic attraction as well as the increased steric and electrostatic repulsions upon the binding of surfactants with zein molecules. Usually, other investigators observed the constant particle size of zein nanoparticles stabilized by the high amount of conventional surfactants.^9^ However, when *C*_NaTC_ increases from 0.24 to 0.4 g L^−1^, while the turbidity of zein/NaTC nanoparticles increases rapidly, there is a much marked increase in the particle size of zein/NaTC nanoparticles, increasing from 76 nm at 0.24 g L^−1^ NaTC to 137 nm at 0.4 g L^−1^ NaTC. SEM pictures show that zein/NaTC nanoparticles at 0.24–0.4 g L^−1^ NaTC possess distinct ellipsoidal shape and big particle size. At *C*_NaTC_ = 0.5 and 0.6 g L^−1^, the samples of zein/NaTC nanoparticles show the high turbidity even with some white precipitates. Thus, the particle size could not be reliably measured at these two NaTC concentrations.

**Fig. 2:**
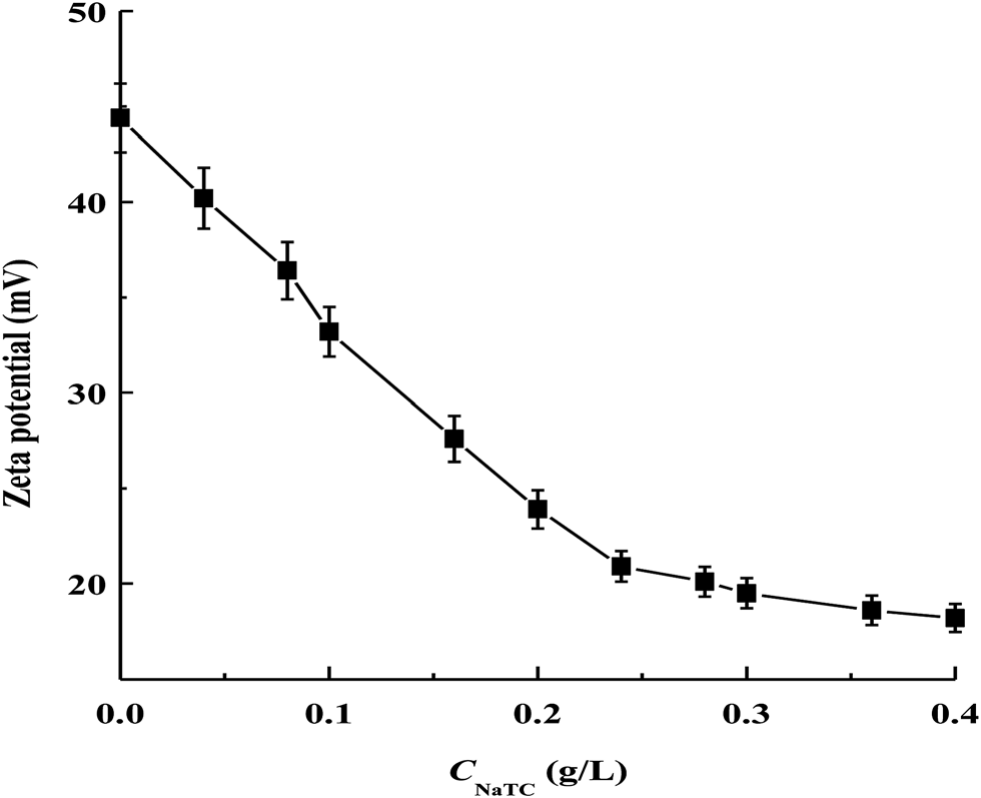
Zeta potential of zein/NaTC nanoparticles as a function of NaTC concentration.

As shown in Fig. 2, the magnitude of zeta potential of zein/NaTC nanoparticles decreases almost linearly from 44 to 21 mV as *C*_NaTC_ increases from 0 to 0.24 g L^−1^. This result can be attributed to the specifically electrostatic binding of the negatively charged SO_3_^−^ group of NaTC molecules with the positively charged NH_3_^+^ groups of zein molecules. Bordbar *et al.* considered that the electrostatic binding sites of protein begin to be occupied by ionic surfactant before the hydrophobic binding due to the higher affinity of electrostatic binding sites than the hydrophobic ones.^20^ Dai *et al.* disclosed the existence of electrostatic interaction between zein and lecithin in the formation of zein/lecithin nanoparticles by measuring the shift of fourier transform infrared bands of amide I and amide II groups of zein.^21^ While the SO_3_^−^ group of NaTC molecules are electrostatically bound with the NH_3_^+^ groups of zein molecules, the flat conformation of NaTC molecules may exert high steric repulsion among zein/NaTC complexes. Thus, zein/NaTC complexes carrying NaTC monomers electrostatically bound may aggregate into smaller zein/NaTC nanoparticles at *C*_NaTC_ = 0–0.24 g L^−1^ compared to plain zein nanoparticles. Our result may be supported by the work from Winuprasith *et al.*,^22^ who found that the addition of small amount of bile salts can stabilize the particle diameter of β-lactoglobulin coated on the surface of gold nanoparticles.

Nevertheless, it is noted that the increase of *C*_NaTC_ from 0.24 to 0.4 g L^−1^ has small influence on the zeta potential of zein/NaTC nanoparticles. Thus, different from the electrostatic binding of NaTC monomers with zein molecules at *C*_NaTC_ = 0–0.24 g L^−1^, there must be another attractive interaction force above 0.24 g L^−1^ NaTC. In fact, bile salt molecules are apt to form dimers through the association between their hydrophobic faces.^13^ Beyond *C*_NaTC_ = 0.24 g L^−1^, the electrostatic binding sites of zein molecules may be almost bound with the headgroup of NaC molecules. Thus, driven by the hydrophobic interaction between the hydrophobic faces of NaTC molecules, the added NaTC molecules may bind with NaTC molecules that already bound to form NaTC dimers on zein polypeptide chains. The generation of NaTC dimers on zein polypeptide chains can cause zein/NaTC complexes to occur more aggregation to form larger zein/NaTC nanoparticles at *C*_NaTC_ = 0.24–0.4 g L^−1^. At *C*_NaTC_ = 0.5 and 0.6 g L^−1^, high order NaTC oligomers may induce the extensive aggregation of zein/NaTC complexes. The discussion about zein/NaTC complexes with different kinds of NaTC aggregates is supported by three ranges of NaTC concentrations in the turbidity result. It is seen that 0.5 g L^−1^ NaTC is about twice 0.24 g L^−1^ NaTC, which indicates the concentration range (0.24–0.5 g L^−1^ NaTC) for the formation of NaTC dimers on zein polypeptide chains.

**Fig. 3:**
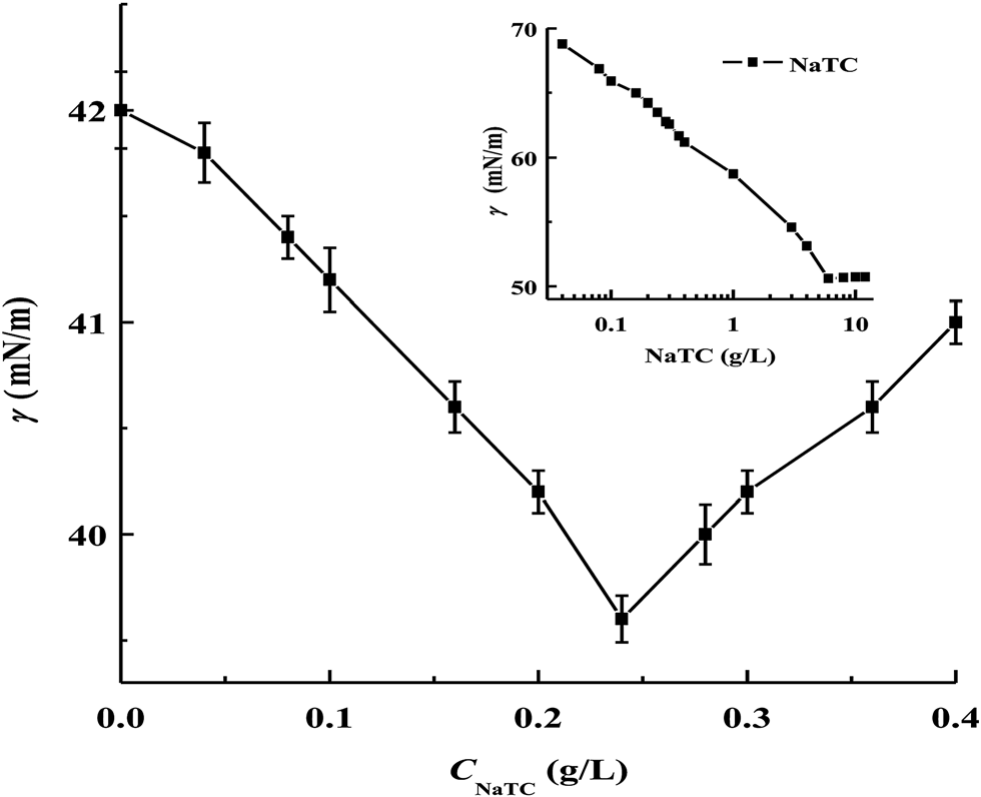
Variations of the surface tension values of zein/NaTC nanoparticles and pure NaTC (inserted figure) as a function of NaTC concentration.

The surface tension measurement can provide more information to understand the formation of zein/NaTC nanoparticles. Fig. 3 gives the surface tension of zein/NaTC nanoparticles as a function of *C*_NaTC_, together with the surface tension of pure NaTC. The critical micelle concentration of pure NaTC determined from its surface tension curve is 6 g L^−1^, which is close to the value reported by Meyerhoffer *et al.* through the fluorescence measurement.^23^ Meanwhile, plain zein nanoparticles have a surface tension value of 42 mN m^−1^, which is smaller than that of pure NaTC at *C*_NaTC_ below or above the critical micelle concentration, which indicates higher surface activity of plain zein nanoparticles than pure NaTC.^24^ Patel *et al.*^8^ and McClements *et al.*^9^ thought that surfactant molecules generate the stabilizer layer around the core of zein nanoparticles, which brings about the reduced surface hydrophobicity, increased electrostatic and steric repulsions. According to this core–shell structure model, zein/NaTC nanoparticles are expected to have higher or same surface tension values compared to plain zein nanoparticles due to lower surface activity of pure NaTC. However, zein/NaTC nanoparticles exhibit the decreased surface tension in the investigated *C*_NaTC_ of 0–0.4 g L^−1^, which disagrees with the binding of NaTC molecules around the core of zein nanoparticles. In the studies of the solubilization of zein, Somasundaran *et al.*^25^ and Mehta *et al.*^26^ found that surfactants can directly bind with zein molecules to form zein/surfactant complexes. Similarly, when NaTC with high water solubility and rapid diffusion is used as the stabilizer, NaTC molecules may preferentially bind with zein molecules to form zein/NaTC complexes. These zein/NaTC complexes may further aggregate into zein/NaTC nanoparticles due to the hydrophobic attraction, like the formation of plain zein nanoparticles by the aggregation of zein molecules.

As shown in Fig. 3, it is noted that the surface tension of zein/NaTC nanoparticles has the contrary change below and above *C*_NaTC_ = 0.24 g L^−1^, which is consistent with the particle size result. The appearance of a minimum value in the surface tension curve of zein/NaTC nanoparticles is in agreement with the work from Maikokera *et al.*^27^ With increasing *C*_NaTC_ from 0 to 0.24 g L^−1^, the decrease in the surface tension of zein/NaTC nanoparticles indicates that zein molecules carrying NaTC monomers electrostatically bound tend to form higher surface-active zein/NaTC nanoparticles. The hydrophilic surface and negatively charged group of NaTC molecules contacting with water phase can increase the hydrophilicity of zein/NaTC nanoparticles, which have high tendency to adsorb at the air/solution interface to decrease the interfacial free energy. However, at *C*_NaTC_ = 0.24–0.4 g L^−1^, the increased surface tension indicates the weakened surface activity of bigger zein/NaTC nanoparticles, which are formed by more aggregation of zein/NaTC complexes carrying NaTC dimers. Stenstam *et al.* also observed that the addition of high amount of SDS into lysozyme causes an increase in the surface tension, indicating the formation of big lysozyme/SDS nanoparticles.^28^

### Tertiary structure of zein molecules in zein/NaTC nanoparticles

**Fig. 4:**
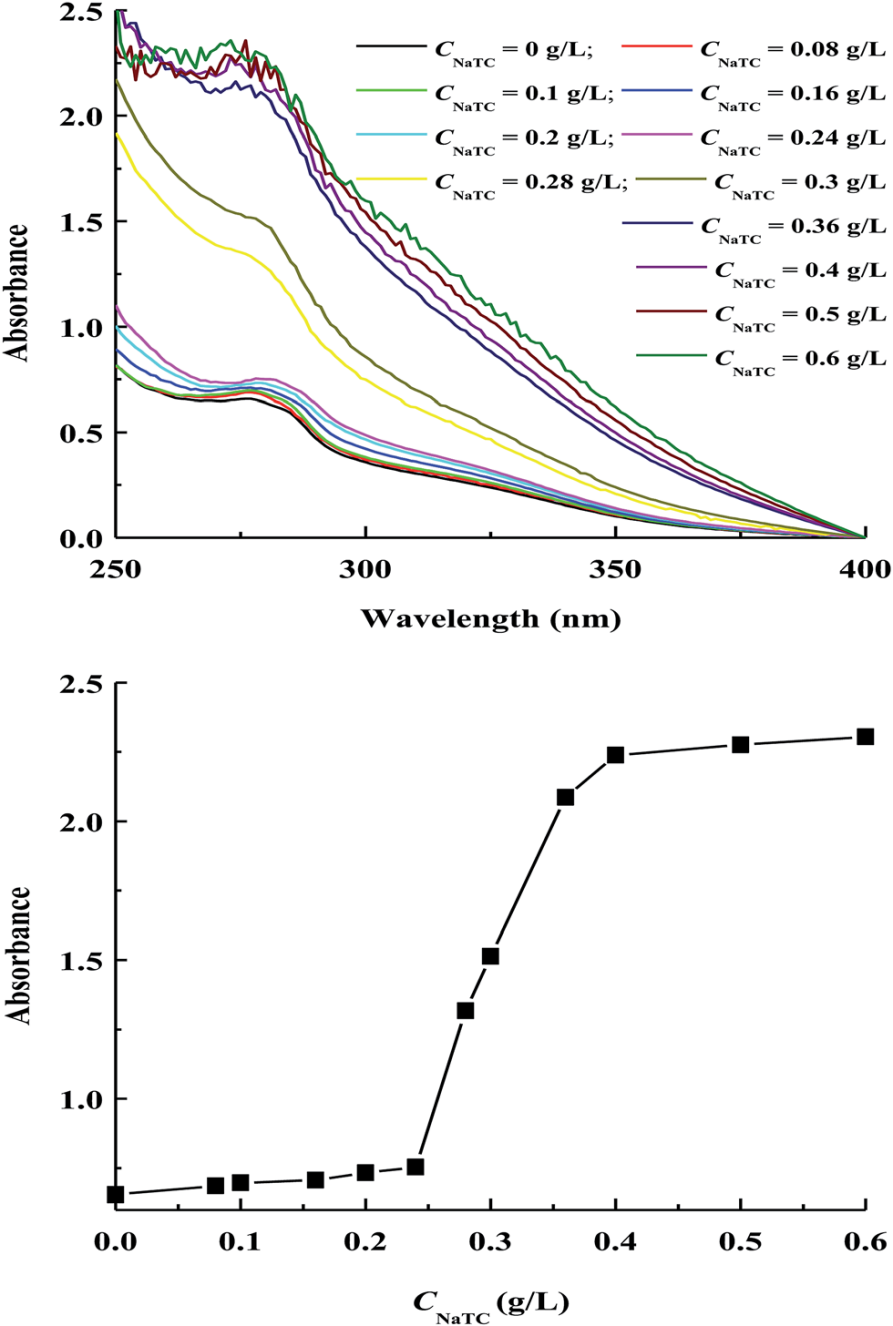
Variations of absorption spectra and absorbance maximum intensity of zein molecules in zein/Tween-20 nanoparticles as a function of NaTC concentration.

The change in the tertiary structure of proteins can be monitored by measuring the absorption and fluorescence spectra due to the presence of benzene-containing chromophores including phenylalanine, tryptophan and tyrosine residues.^26^ Zein molecules contain a high level of tyrosine residues (∼5.1 wt%), while the levels of phenylalanine and tryptophan residues are insignificant.^5^Fig. 4 shows the variations of absorption spectra and absorbance maximum intensity of zein molecules in zein/NaTC nanoparticles as a function of *C*_NaTC_. The characteristic peak around 278 nm is mainly resulted from the absorbance by tyrosine residues of zein molecules.

It is noteworthy that the absorbance maximum intensity of zein molecules exhibits as the sigmoidal shape plot as a function of *C*_NaTC_, including an initial minor increase, and an appreciable increase before reaching a final plateau. This sigmoidal absorbance plot indicates the conformational transition of zein molecules from folded to unfolded structure according to the two state mechanism for protein denaturation.^26,29^ Deo *et al.* previously found that the tertiary structure of zein molecules does not change measurably if small amount of SDS is added.^19^ At *C*_NaTC_ = 0–0.24 g L^−1^, the electrostatic interaction between the negatively headgroup of NaTC monomers and the positively charged groups of zein molecules is expected to have little disturbance to the compact conformation of zein molecules. At *C*_NaTC_ = 0.24–0.4 g L^−1^, the rapid increase in the absorbance is perhaps driven by the formation of NaTC dimers due to the hydrophobic interaction between the convex hydrophobic surface of NaTC molecules. On one hand, the formation of NaTC dimers can lead to the partial unfolding of zein polypeptide chains with the exposure of more tyrosine residues. On the other hand, NaTC dimers can provide enhanced hydrophobic microenvironment for tyrosine residues owing to the large and planar hydrophobic moiety of NaTC molecules. Therefore, the addition of NaTC may cause zein molecules in zein/NaTC nanoparticles to give a rapid increase in the absorbance at *C*_NaTC_ = 0.24–0.4 g L^−1^. Similar result was previously reported by Rafati *et al.* for the conformation change of bovine serum albumin with a series of cationic surfactants.^30^ Additionally, unfolding of zein molecules appears to be completed beyond 0.4 g L^−1^ NaTC, indicated by the nearly unchanged absorbance of zein/NaTC nanoparticles at *C*_NaTC_ = 0.5–0.6 g L^−1^.

**Fig. 5:**
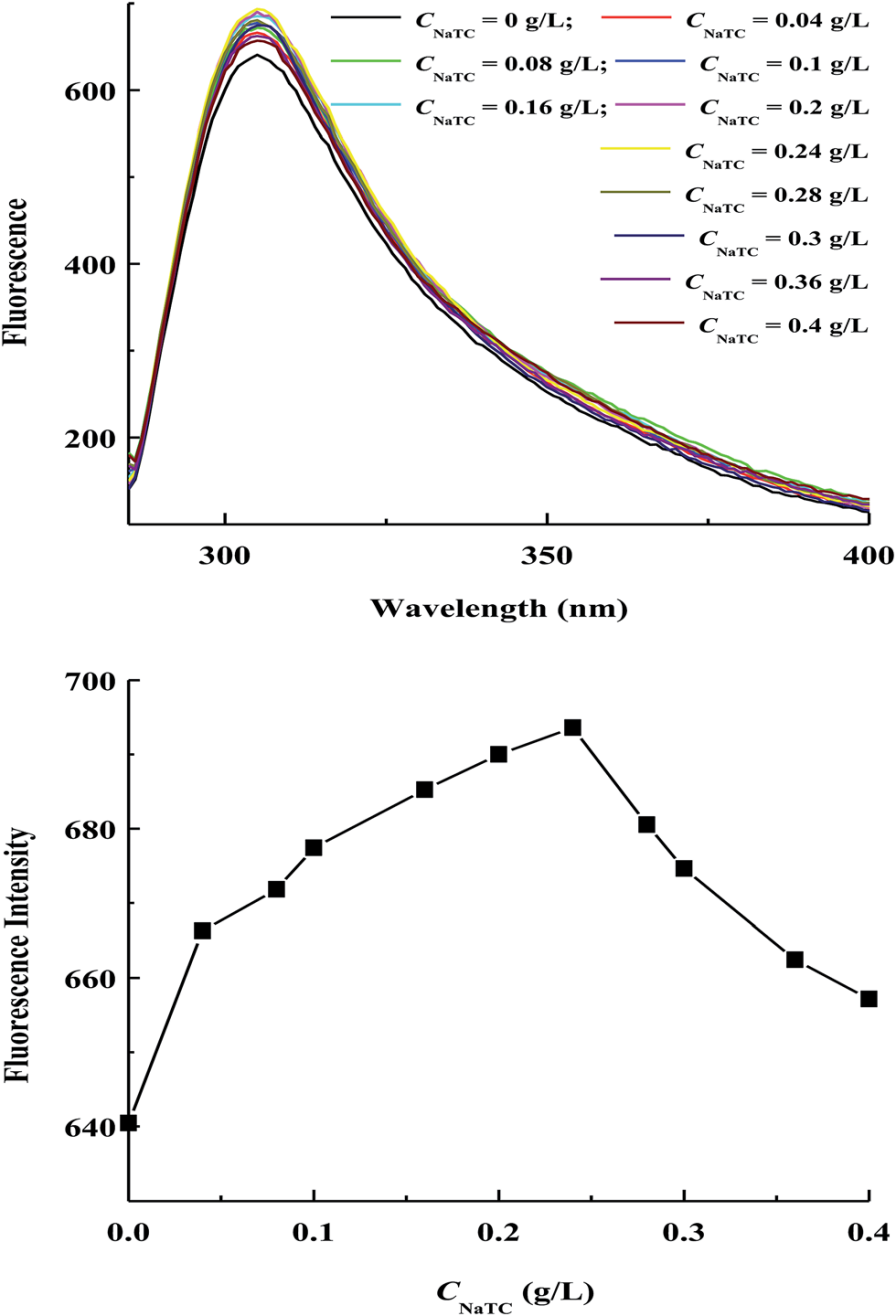
Variations of fluorescence spectra and fluorescence maximum intensity of zein molecules in zein/NaTC nanoparticles as a function of NaTC concentration.

Fig. 5 gives the fluorescence spectra and fluorescence maximum intensity of zein molecules in zein/NaTC nanoparticles as a function of *C*_NaTC_. All samples exhibit a strong fluorescence emission peak around 304 nm after being excited at 278 nm, which could be mainly attributed to the unique property of tyrosine residues of zein molecules. The fluorescence maximum intensity of zein molecules goes through a maximum at *C*_NaTC_ = 0.24 g L^−1^ in the range of 0–0.4 g L^−1^ NaTC. This result is consistent with the work from Maikokera *et al.*,^27^ who found that low concentration of SDS results in a large increase in the fluorescence intensity of the protein extracted from *Moringa oleifera* seeds, which begins to decrease at high concentration of SDS. At *C*_NaTC_ = 0–0.24 g L^−1^, the above absorption measurement reveals that the electrostatic interaction between the negatively headgroup of NaTC monomers and the positively charged groups of zein molecules is a significant force, which has little influence on the compact conformation of zein molecules. However, the special molecular structure of NaTC is relatively flat, which tends to lie flat on the surface of zein molecules.^22^ Because of the incomplete separation of the hydrophobic and hydrophilic parts of NaTC molecules, the hydrophobic face of NaTC monomers may have somewhat interaction with the adjacent hydrophobic groups of zein molecules. This hydrophobic interaction may result in an altered microenvironment around the tyrosine residues and an enhancement of zein fluorescence.^10^ On the other hand, the decreasing of zein fluorescence at *C*_NaTC_ = 0.24–0.4 g L^−1^ can be ascribed to the unfolding of zein molecules induced by the formation of NaTC dimers on zein polypeptide chains. The opening of hydrophobic pockets of zein molecules causes the tyrosine residues to contact with the quencher molecules of water and oxygen.^14,31^ Moreover, the loss of native structure and the partially unfolded state of zein molecules can induce a high propensity to undergo aggregation owing to the conformation rearrangement with the exposure of more hydrophobic groups,^3^ which is indicated by bigger particle size of zein/NaTC nanoparticles above 0.24 g L^−1^ NaTC.

### Secondary structure of zein molecules in zein/NaTC nanoparticles

**Fig. 6:**
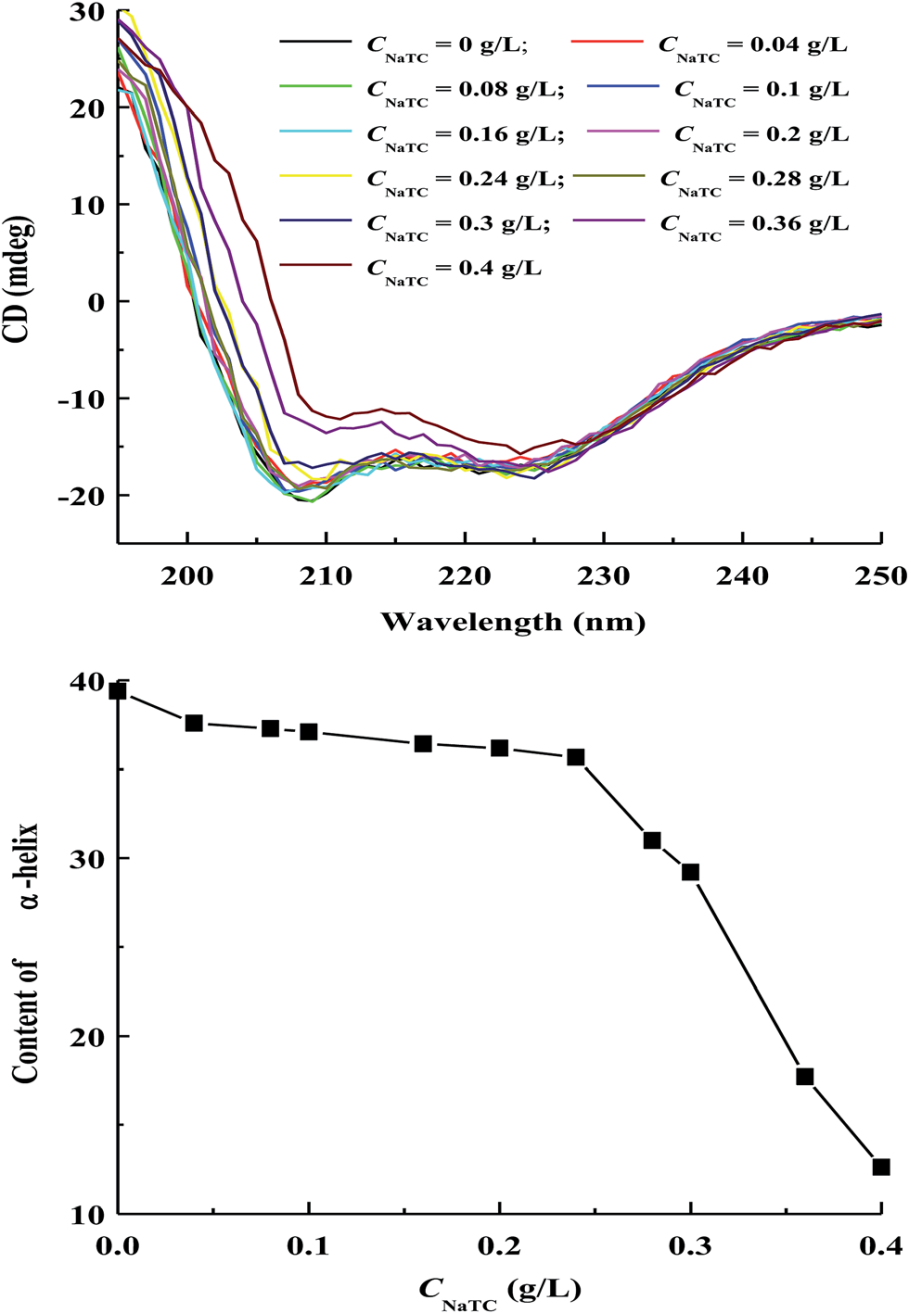
Variations of CD spectra and α-helix content of zein molecules in zein/NaTC nanoparticles as a function of NaTC concentration.

Circular dichroism (CD) measurement can be carried out for the characterization of the secondary structure changes of zein molecules in zein/NaTC nanoparticles.^32,33^ As shown in Fig. 6, CD spectra of zein molecules in zein/NaTC nanoparticles give two negative peaks at 208 and 222 nm, corresponding to the helical conformation of zein molecules.^34^ The negative troughs at 208 and 222 nm are seen to collapse toward less negative values with increasing *C*_NaTC_, which reveals the loss in α-helix content of zein molecules upon the binding of NaTC molecules. The change of α-helix content of zein molecules as a function of *C*_NaTC_ is also included in Fig. 6, which is quantitatively estimated from two negative peaks of CD spectra. In plain zein nanoparticles, the α-helix content of zein molecules is 39%, which is in agreement with the value reported by other people.^5^ When mixing of surfactants with water-soluble bovine serum albumin, Ahluwalia *et al.*^31^ and Li *et al.*^35^ found that the added surfactants could give protection or enhancement of the helical structure of protein. However, the addition of NaTC brings about the reduction of molar ellipticity and α-helix amount of zein molecules in zein/NaTC nanoparticles. This result indicates that the binding of NaTC molecules may destroy the hydrogen bonding between the polypeptide chains of zein molecules, leading to the decrease in the fraction of helical structure of zein molecules.^36^

Consistent with the tertiary structure change of zein molecules, the change of α-helix content of zein molecules is significantly dependent on NaTC concentration. With increasing *C*_NaTC_, the content of α-helix initially has a slow decrease before 0.24 g L^−1^ NaTC, but decreases distinctly at 0.24–0.4 g L^−1^. When *C*_NaTC_ is lower than 0.24 g L^−1^, our result reveals that the secondary structure of zein molecules is little influenced by the electrostatic binding of NaTC monomers. When *C*_NaTC_ increases from 0.24 to 0.4 g L^−1^, the sharp decreasing of α-helix content of zein molecules suggests that the formation of NaTC dimers due to the hydrophobic interaction may greatly destroy the helical structure of zein molecules.^9,26^

## Conclusion

**Fig. 7:**
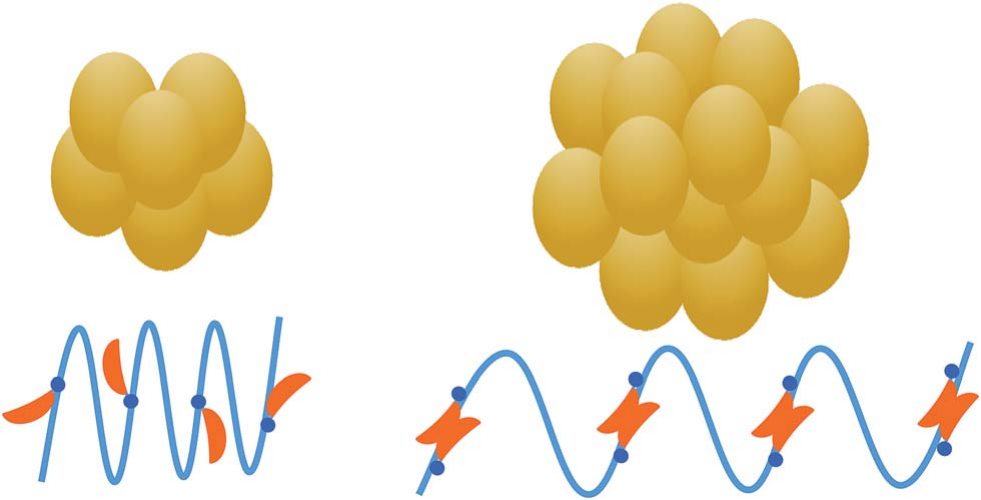
Schematic representation of the formation mechanism of zein/NaTC nanoparticles at different NaTC concentrations (left: *C*_NaTC_ = 0–0.24 g L^−1^; right: *C*_NaTC_ = 0.24–0.4 g L^−1^).

This work has revealed the existence of a critical NaTC concentration for the interaction and conformation changes of zein with NaTC in zein/NaTC nanoparticles. Zein/NaTC nanoparticles are formed by the aggregation of zein/NaTC complexes, which have different structures at low and high NaTC concentrations. Fig. 7 gives a schematic illustration for the formation mechanism of zein/NaTC nanoparticles at different NaTC concentrations. At *C*_NaTC_ = 0–0.24 g L^−1^, zein/NaTC nanoparticles of decreased particle size are formed by the aggregation of zein/NaTC complexes carrying NaTC monomers electrostatically bound. At *C*_NaTC_ = 0.24–0.4 g L^−1^, the formation of NaTC dimers on zein polypeptide chains due to the hydrophobic interaction causes zein/NaTC complexes to occur more aggregation into larger zein/NaTC nanoparticles. The spectrometric measurements show that zein molecules have little conformation changes at low NaTC concentrations, whereas the addition of high amount of NaTC can greatly promote the unfolding of zein molecules and destroy the α-helical structure. Zein nanoparticles, holding the attractive advantages of inexpensive, less allergenic, more biocompatible and biodegradable, have currently received much attention for the encapsulation of drug and bioactive molecules. As the biosurfactants extensively distributed in human gastrointestinal tract, bile salts may significantly influence the biological fate of zein nanoparticles and encapsulated bioactive molecules. Therefore, the knowledge of the interaction between bile salts and zein nanoparticles has important values for the development of zein-based systems with beneficial functions for the human's health.

## Conflicts of interest

There are no conflicts to declare.

## Supplementary Material
